# Kutane Leishmaniose fehlgedeutet als epithelialer Hautkrebs

**DOI:** 10.1007/s00105-025-05473-5

**Published:** 2025-02-05

**Authors:** Ana-Lee Gerdes, Alexander Kreuter, Uwe Hillen, Jörg Schaller, Frank Oellig, Julia Hyun, Valentina Laura Müller

**Affiliations:** 1 Klinik für Dermatologie, Venerologie und Allergologie, Helios St. Johannes Klinikum Duisburg, Dieselstraße 185, 47166 Duisburg, Deutschland; 2 Klinik für Dermatologie, Venerologie und Allergologie, Helios St. Elisabeth Klinik Oberhausen, Universität Witten/Herdecke, Oberhausen, Deutschland; 3 Dermatopathologie Duisburg Essen, Essen, Deutschland; 4 Pathologisches Institut, Mülheim an der Ruhr, Deutschland

**Keywords:** Kutane Leishmaniose, Plattenepithelkarzinom, Sandmücke, *Leishmania infantum*, Photodynamische Therapie, Cutaneous leishmaniosis, Squamous cell carcinoma, Sand fly, *Leishmania infantum*, Photodynamic therapy

## Abstract

Die durch Sandmücken der Gattung *Phlebotomus* übertragene kutane Leishmaniose gehört zu den bedeutendsten Reisedermatosen in Deutschland. In den vergangenen Jahren konnte eine vermehrte Zunahme der Erkrankung bei Reiserückkehrern aus endemischen Gebieten, zu denen unter anderem die Mittelmeerregion mit Spanien und Italien zählt, beobachtet werden. In diesen Regionen ist *Leishmania infantum* des Leishmania-donovani-Komplexes der am häufigsten nachweisbare Erreger. Wir berichten von 2 durch *Leishmania infantum* verursachten Fällen einer kutanen Leishmaniose mit solitären bzw. multilokulären Läsionen, die initial als aktinische Keratosen bzw. Plattenepithelkarzinome fehldiagnostiziert wurden.

## Abstract (en)

Cutaneous leishmaniasis, transmitted by sand flies of the genus *Phlebotomus*, is one of the most important traveler’s dermatoses in Germany. In recent years, an increased incidence of the disease has been observed in travelers returning from endemic areas, including the Mediterranean region of Spain and Italy. In these regions, *Leishmania infantum* belonging to the *Leishmania donovani* complex is characteristically the most frequently detected pathogen. We herein report two cases of cutaneous leishmaniasis caused by *Leishmania infantum* with solitary or multilocular lesions, initially misdiagnosed as actinic keratosis and cutaneous squamous cell carcinoma.

Die kutane Leishmaniose (CL) ist als parasitäre Erkrankung mit endemischem Vorkommen v. a. in Afrika, Asien und Südamerika weltweit bekannt. Aufgrund globaler klimatischer Veränderungen in den vergangenen Jahren fühlt sich die Sandmücke *Phlebotomus perniciosus* als Überträger protozoischer Parasiten der Gattung *Leishmania* auch in europäischen Breitengraden zunehmend heimisch [1, 9]. Zahlreiche Endemiegebiete mit typischerweise Nachweis von *L. infantum* sind im Mittelmeerraum vorhanden. Zunehmend wird die CL aus beliebten spanischen Urlaubsregionen wie der balearischen Insel Mallorca sowie südlichen und westlichen Küstenregionen Italiens als Reisedermatose nach Deutschland importiert [2]. Hierzulande bleibt die CL aufgrund der klinisch häufig unspezifischen Erscheinungsformen sowie langen Inkubationszeiten insbesondere bei Reiserückkehrern aus endemischen Gebieten nicht selten unerkannt und wird erst nach langem Bestehen diagnostiziert und therapiert [3]. Wir berichten über 2 Patientenfälle, die unter dem initialen Verdacht auf keratinozytischen Hautkrebs zu uns geschickt wurden und deren Diagnostik letztendlich den Zufallsbefund einer CL erbracht hat.

## Fallbericht 1

**Abb. 1:**
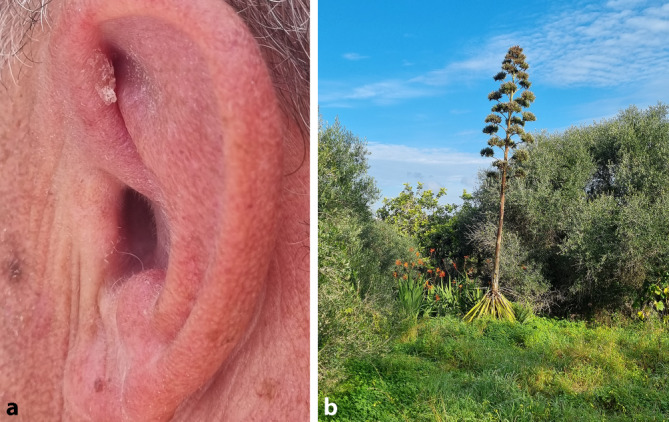
Klinische Präsentation von Patient 1 bei Erstvorstellung sowie später nach Diagnosestellung anhand mitgebrachter Fotografien. **a** Umschriebene, ca. 0,8 × 0,5 cm durchmessende, hyperkeratotische, erythematöse Plaque an der rechten Ohrhelix; **b** Garten der Finca auf Mallorca (südliche Küstenregion), dem Urlaubsdomizil des Patienten

**Abb. 2:**
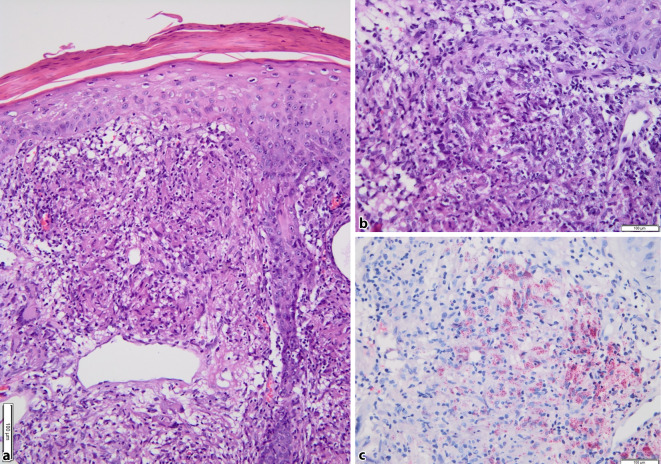
Histopathologische Befunde der Biopsie aus der Ohrmuschel links. **a** Diffuses, tief reichendes Entzündungsinfiltrat (Hämatoxylin & Eosin); **b** Vorhandensein massiver Amastigoten in der Standardfärbung mit Hämatoxylin & Eosin. **c** Bestätigung der Amastigoten in der immunhistochemischen Spezialfärbung (MTB1-Färbung)

Ein 56-jähriger Patient wurde zur Hautkrebsvorsorge in unserer Ambulanz vorstellig. Vorerkrankungen waren nicht bekannt. Eine Dauermedikation bestand zum damaligen Zeitpunkt nicht. Allergien wurden verneint. In der körperlichen Untersuchung zeigte sich ein nahezu blander Hautbefund. Es imponierte jedoch im Bereich der linken Ohrhelix eine umschriebene, ca. 0,8 × 0,5 cm durchmessende, hyperkeratotische, erythematöse Plaque, die dem Patienten erstmalig wenige Wochen nach einem Fincaurlaub auf Mallorca aufgefallen war (Abb. 1a, b). Ein Insektenstich war nicht erinnerlich. Zum Ausschluss eines Morbus Bowen bzw. eines initialen Plattenepithelkarzinoms erfolgte eine Probebiopsie. Die histologische Befundung zeigte eine breit parakeratotisch verhornte Epidermis mit Serumeinschlüssen, eine ausgedehnte Entzündungsreaktion mit epitheloidzelligen Granulomen und Fremdkörperriesenzellen unterhalb der Epidermis sowie zahlreiche Amastigoten (Abb. 2a–c). In der molekularpathologischen Untersuchung konnte mittels PCR-Diagnostik L.-donovani-Komplex-assoziierte DNS (Desoxyribonukleinsäure) isoliert werden. Eine Sequenzierung zur exakten Speziesbestimmung gelang jedoch nicht. Unter Berücksichtigung der Reiseanamnese (Mallorca) war am ehesten von einer Infektion mit *L. infantum* auszugehen [12]. Therapeutisch entschieden wir uns für die Einleitung einer photodynamischen Therapie mittels Tageslicht (Daylight-PDT [DL-PDT]). Das betroffene Hautareal wurde nach vorheriger Applikation von Delta-Aminolävulinsäure (δ-ALA) unter Okklusion für 1 h mit Tageslicht 2‑mal wöchentlich über einen 4‑wöchigen Zeitraum bestrahlt. Hierunter zeigte sich die Hautveränderung vollständig regredient. Ein Rezidiv ist im 1‑Jahres-Follow-up nicht aufgetreten.

## Fallbericht 2

**Abb. 3:**
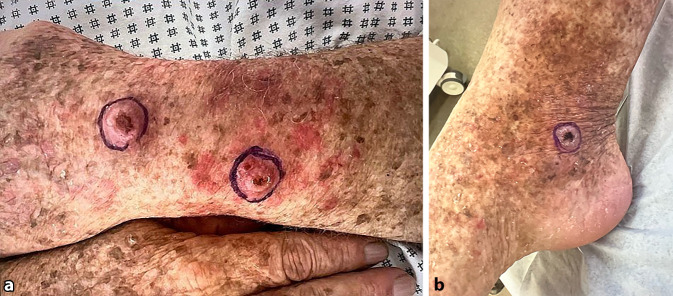
Klinische Präsentation von Patient 2 bei Erstvorstellung. **a** Zwei umschriebene, jeweils ca. 1,5 cm durchmessende, zentral ulzerierte Nodi mit aufgeworfenem Randsaum am linken Unterarm. **b** Klinisch vergleichbarer Befund am rechten Malleolus medialis

**Abb. 4:**
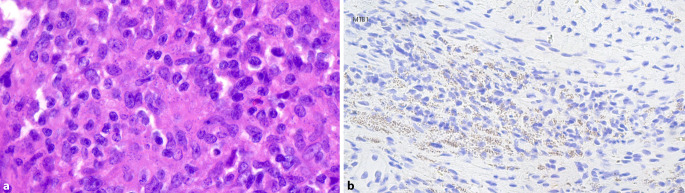
Histopathologische Befunde der Exzision einer Läsion am linken Unterarm. **a** Verdacht auf das Vorhandensein von Amastigoten in der Standardfärbung mit Hämatoxylin & Eosin (Hämatoxylin & Eosin, Originalvergrößerung 200:1). **b** Bestätigung der Amastigoten in der immunhistochemischen Spezialfärbung (MTB1-Färbung, Originalvergrößerung 100:1)

Ein 68-jähriger Patient wurde mit seit ca. 2 Jahren bestehenden, größenprogredienten Nodi an der linken distalen, streckseitigen oberen Extremität und am rechten Malleolus medialis in unserer dermatologischen Ambulanz vorstellig. Der Patient war aufgrund einer Feldkanzerisierung nach jahrelanger Psoriasis-bedingter PUVA-Therapie sowie erhöhter UV-Exposition durch Outdoor-Aktivitäten in unserer Klinik bereits bekannt. Mehrfach waren bereits in der Vergangenheit epitheliale Hauttumoren an diversen Lokalisationen exzidiert worden. Im Rahmen der aktuellen Untersuchung zeigten sich insgesamt 2 unscharf begrenzte, jeweils ca. 1,5 cm durchmessende, zentral ulzerierte, erythematöse Nodi mit aufgeworfenem Randsaum am linken streckseitigen Unterarm sowie ein vergleichbarer Befund am rechten Malleolus medialis (Abb. 3a, b). Im Bereich des restlichen Integuments fand sich eine ausgeprägte UV-induzierte Poikilodermie mit PUVA-induziertem UV-Schaden (sog. „PUVA-Freckling“). Die in toto erfolgte Exzision der Hauttumoren konnte den initialen Verdacht dreier Plattenepithelkarzinome nicht bestätigen. Histopathologisch fanden sich stattdessen fibrinbelegte Ulzerationen mit diskreter Akanthose des Plattenepithels im Randbereich sowie knotige, lymphohistiozytäre Infiltrate mit basophilen Niederschlägen mit CD1a-Positivität (Abb. 4a, b). Eine PCR-Diagnostik erbrachte den Nachweis eines L.-donovani-Komplexes. Die weiterführende Sequenzierung ergab *L. infantum*. Auf erneute Nachfrage gab der Patient einen mehrwöchigen Camping-Urlaub in der Toskana vor dem ersten Auftreten der Hautläsionen an. Therapeutisch planten wir eine, wie in Fallbericht 1 beschriebene, additive DL-PDT, die jedoch seitens des Patienten abgelehnt wurde. Eine Nachkontrolle war bei „Lost to follow up“-Situation nicht möglich.

## Diskussion

Die CL stellt eine der am häufigsten aus beliebten Urlaubsregionen rund um das Mittelmeer nach Deutschland importierten Reisedermatosen dar. Eine zunehmende Inzidenz von Fällen aus südeuropäischen Endemiegebieten wie Mallorca und westlichen bzw. südlichen Regionen Italiens ist zu beobachten [7, 8]. Im Mittelmeerraum sind 4 *Leishmania*-Spezies nachgewiesen worden, bei denen es sich um Protozoenparasiten der Alten Welt (AW) handelt [2, 11]. Verbreitung findet hierbei insbesondere der in periurbanen bzw. ländlichen Urlaubsgebieten dominierende parasitäre Erreger *L. infantum* des L.-donovani-Komplexes durch die dämmerungs- und nachtaktive Sandmücke der Gattung *Phlebotomus*. *Phlebotomus perniciosus* ist ein sowohl in Spanien als auch Italien vorkommender, kompetenter Vektor mit Haushunden und Nagetieren als primärem Erregerreservoir sowie Menschen als akzidentiellem Wirt [9]. Eine Ausbreitung des Vektors außerhalb endemischer Gebiete ist aufgrund von Klimawandel, globaler Erwärmung, Umweltveränderungen sowie vermehrter Migration zunehmend häufiger [9]. Die Diagnosestellung kann durch eine variable Klinik mit häufig atypischen Verlaufsformen, unspezifischer Symptomatik sowie einer oftmals lückenhaften oder fehlenden Reiseanamnese erschwert sein [2, 10]. Nicht selten werden die CL als epitheliale Hauttumoren wie in unseren beiden Patientenfällen, Pseudolymphome, tiefe Mykosen, Ekthymata, bakterielle Hautinfektionen durch *Staphylococcus aureus* oder β‑hämolysierende Streptokokken, atypische Mykobakteriosen, Sarkoidose, Lepra oder Syphilis fehldiagnostiziert [5, 9]. Aus diesem Grund sind für die histologische Diagnostik adäquate Probenentnahmen unabdinglich. Insbesondere bei Flachexzidaten mit nur oberflächlicher Bindegewebserfassung kann die pseudoepitheliomatöse Hyperplasie histologisch falsch interpretiert werden und zur Fehldiagnose eines gut differenzierten spinozellulären Karzinoms führen. Die fatale Konsequenz ist die komplette Exzision, an der dann letztendlich erst die richtige Diagnose einer Leishmaniose gestellt wird. Sekundärinfektionen sowie „Mistreatment“ können das Resultat einer Fehldiagnose unterstützen, was folglich durch Einleitung inadäquater Therapieansätze zu einer erhöhten Morbidität und verminderten Lebensqualität führt [3]. Diagnostisch beweisend ist der histo- und molekularpathologische Nachweis von Leishmanien oder Leishmanien-DNS im Gewebe [1]. Chronische Krankheitsverläufe erschweren durch Auftreten granulomatöser entzündlicher Infiltrate sowie reduzierte Anzahl parasitierter Makrophagen oftmals den Erregernachweis [10]. Die PCR-Sequenzierung stellt aufgrund hoher Sensitivität und Spezifität den diagnostischen Goldstandard dar und dient zur exakten Speziesdifferenzierung vor Auswahl eines geeigneten Therapieregimes [1]. Alternativ kann sich die Therapieentscheidung in Ausnahmefällen, wie bei unserem Patientenfall 1, an epidemiologischen Daten aus der mit *Leishmania*-Spezies potenziell infizierten Urlaubsregion orientieren. In der zuletzt im November 2010 aktualisierten AWMF-Leitlinie zur Diagnostik und Therapie der kutanen und mukokutanen Leishmaniose sind diverse topische und systemische Therapieansätze abhängig von der diagnostizierten *Leishmania*-Spezies beschrieben [12]. Grundsätzlich sollte bei Nachweis von *L. infantum* in immunsupprimierten Patienten zunächst eine extrakutane bzw. viszerale Beteiligung ausgeschlossen werden [12]. Diesbezüglich kann zum Ausschluss einer disseminierten Infektion eine Knochenmarkbiopsie mit Erregerdiagnostik bei bestehender Immundefizienz erwogen werden [5]. Ein selbstlimitierender Krankheitsverlauf mit narbiger Ausheilung sowie Immunisierung ist bei durch *L. infantum* ausgelöster CL spätestens nach ca. 2 Jahren möglich [2, 3]. Zur Senkung des Risikos irreversibler, kosmetisch-ästhetischer Komplikationen ist jedoch ein vorzeitiges therapeutisches Vorgehen anzuraten [2, 3]. Periläsionales Antimon, häufig in Kombination mit Kryotherapie, ist ein etabliertes Therapieverfahren bei klinisch einfachen Hautläsionen [2]. Komplexe Läsionen (> 3 Läsionen, eine Einzelläsion > 40 mm Durchmesser, Läsionen im Gesicht, an Händen, Gelenken oder Haut-Schleimhaut-Übergängen, Satellitenläsionen, Lymphangitis oder -adenitis) sollten systemisch mit Antimon, Miltefosin oder liposomalem Amphotericin B behandelt werden [2, 13, 14]. Als eine in der Leitlinie beschriebene weitere Therapieoption ist bei unkomplizierter CL der Off-label-Einsatz einer PDT mit Rotlicht (cPDT) oder DL-PDT angegeben [12]. In der Literatur finden sich mehrere Fälle einer durch *L. major, L. tropica* oder *L. donovani* verursachten CL mit erfolgreichem Therapieansprechen [1]. Für DL-PDT und cPDT ist eine gleich gute Wirksamkeit belegt [4]. *L. infantum*-assoziierte PDT-Ansätze sind bislang nicht verzeichnet. Umso interessanter ist der Nachweis einer vollständigen Regredienz unter Initiierung einer erfolgreich empirisch eingesetzten, insgesamt 4‑wöchigen DL-PDT mit 2‑mal wöchentlichen Bestrahlungen einer CL mit unbekanntem Erreger (vermutlich *L. infantum*) in dem beschriebenen Patientenfall 1 unserer Klinik. Der Vorteil der PDT gegenüber den derzeit empfohlenen, topischen und systemischen Maßnahmen besteht in einem effektiven, nebenwirkungsarmen, nichtinvasiven Behandlungsansatz mit kosmetisch vielversprechendem, meist narbenfreiem Resultat [1, 4, 6]. Als mögliche therapielimitierende Faktoren müssen neben dem erhöhten Zeitaufwand (mehrere PDT-Sitzungen) auch Off-label-Use und damit verbundene fehlende Kostenübernahme durch die Krankenkassen erwähnt werden. Zudem können ausgeprägte Schmerzsymptomatik, Entzündungsreaktion oder Hypo- oder Hyperpigmentierung zu einem vorzeitigen Therapieabbruch führen [1]. Daher ist die nebenwirkungsärmere DL-PDT der cPDT potenziell überlegen. Immunsuppression sowie großflächige, tiefe Läsionen werden als mögliche Kontraindikationen bei DL-PDT-Monotherapie betrachtet [4]. Burmann et al. haben basierend auf einem klinischen Therapieerfolg mit mehreren Zyklen einer Rot- bzw. Grünlicht-PDT in 3 Patientenfällen mit CL-Läsionen, verursacht durch *L. major* und *L. tropica*, die PDT als mögliche Monotherapie vorgeschlagen und PDT als Adjuvans bei systemisch behandelten, komplexen Läsionen in der Therapieauswahl erwogen [1]. Diesbezüglich existiert jedoch bei *L. infantum* keine valide Datenlage. Inwieweit die PDT sich zukünftig als zuverlässige und sicher wirksame Therapieempfehlung zunächst in der Monotherapie bei durch *L. infantum* assoziierter CL etablieren lässt, bleibt daher abzuwarten.

## Fazit für die Praxis

- Tropendermatosen wie die CL (z. B. verursacht durch *L. infantum*) sind innerhalb Europas mittlerweile als endemisch zu bewerten.
- Bei entsprechendem Verdacht sollte eine Urlaubsanamnese als essenzieller Bestandteil der Anamnese erhoben werden.
- Insbesondere bei Reiserückkehrern aus Endemiegebieten ist die CL differenzialdiagnostisch von epithelialen Hauttumoren abzugrenzen.
- Die PCR-Sequenzierung als Goldstandard in der Speziesdiagnostik ist Voraussetzung für eine adäquate, Leishmanien-spezifische Therapie.
- Die DL-PDT stellt eine sehr effiziente, schmerz- und nebenwirkungsarme Therapieoption bei solitären Leishmanioseherden dar.
